# Neck Circumference as a Simple Screening Tool for Metabolic Syndrome in Urban Adults: A Community-Based Cross-Sectional Study From Jaipur, India

**DOI:** 10.7759/cureus.108174

**Published:** 2026-05-03

**Authors:** Madan L Moond, Kusum Gaur

**Affiliations:** 1 Department of Preventive and Social Medicine, Sawai Man Singh (SMS) Medical College, Jaipur, IND

**Keywords:** cardiovascular risk, cut-off, diabetes, dyslipidemia, hypertension, metabolic syndrome, neck circumference, obesity, screening tool, urban india

## Abstract

Background: Metabolic syndrome (MetS) is a constellation of cardiometabolic risk factors like abdominal obesity, hypertension, hyperglycemia, and dyslipidemia associated with substantially increased risk of cardiovascular disease (CVD) and type 2 diabetes mellitus (T2DM). Conventional screening methods involving waist circumference (WC) measurements can be challenging in resource-limited settings. Neck circumference (NC) has emerged as a simpler, non-invasive anthropometric marker of upper-body adiposity with potential utility for MetS screening.

Objective: To determine the prevalence of MetS and its sociodemographic correlates in an urban community, to examine the association and correlation of NC with MetS and other anthropometric indices, and to establish optimal NC cut-off values for predicting MetS in adult males and females.

Methods: A community-based cross-sectional study was conducted from April to July 2023 in the field practice area of the Urban Health Training Centre (UHTC) attached to Sawai Man Singh (SMS) Medical College, Jaipur. A total of 400 adults aged 18-60 years were enrolled using systematic random sampling. MetS was diagnosed using the National Heart, Lung, and Blood Institute (NHLBI) criteria (presence of ≥3 of five components). NC was measured at the level of the thyroid cartilage with an inelastic tape. Associations were analyzed using the chi-square test; correlations using Pearson's coefficient; and optimal NC cut-off values using receiver operating characteristic (ROC) curve analysis.

Results: The mean age of participants was 39.11 ± 12.7 years, with a slight female predominance (M: F = 0.93). MetS was present in 43.5% of subjects. Abnormal NC was observed in 33.5% of participants (mean NC: 35.0 ± 3.1 cm). NC was significantly associated with MetS (chi-square = 218.71, p < 0.001); 95.5% of those with abnormal NC had MetS versus 17.3% with normal NC. NC showed strong positive correlations with WC (r = 0.705, p < 0.001) and BMI (r = 0.581, p < 0.001). ROC analysis identified optimal NC cut-off values of 37 cm in males (sensitivity 87.0%, specificity 96.0%, diagnostic accuracy 92.7%) and 34 cm in females (sensitivity 71.4%, specificity 92.2%, diagnostic accuracy 81.6%). Age, sex, education, occupation, socioeconomic status, personal habits, and marital status were significantly associated with MetS.

Conclusions: NC is a reliable, inexpensive, and clinically practical screening tool for MetS in urban Indian adults. Cut-off values of 37 cm for males and 34 cm for females demonstrate excellent and clinically meaningful diagnostic performance, respectively, facilitating population-level MetS screening in primary care settings in Rajasthan and comparable populations.

## Introduction

Metabolic syndrome (MetS) is a clustering of interrelated cardiometabolic risk factors like central obesity, elevated blood pressure, impaired fasting glucose, hypertriglyceridemia, and reduced high-density lipoprotein cholesterol (HDL-C) that substantially increase the risk of cardiovascular disease (CVD), type 2 diabetes mellitus (T2DM), and stroke [1]. The syndrome has become a global public health concern of the highest order, with an estimated one-quarter of the world's adult population meeting diagnostic criteria [2].

In India, the pooled prevalence of MetS ranges from 10% to 50% across diverse regions, with consistently higher rates in urban compared to rural populations [3]. Urban Rajasthan, an economically transitioning region, remains underrepresented in recent MetS burden data. The concurrent rise in sedentary lifestyles, caloric surplus, and psychosocial stressors in Indian cities is accelerating the epidemiological transition toward non-communicable diseases (NCDs), placing enormous strain on already resource-constrained health systems [4].

Early identification of MetS is essential for preventive interventions. Waist circumference (WC), a cornerstone of MetS diagnosis, can be technically challenging to measure accurately in field conditions and may be affected by abdominal contents, clothing, and patient cooperation. Neck circumference (NC), reflecting upper-body subcutaneous adipose tissue, has attracted considerable interest as an alternative or complementary anthropometric marker. Several studies have demonstrated significant associations between NC and MetS components, including insulin resistance, dyslipidemia, hypertension, and central obesity [5-8]. NC is quick to measure, highly reproducible, and not influenced by respiration or meal timing.

Despite growing evidence globally, data on NC as a MetS screening tool in the North Indian urban context are sparse. Published NC cut-off values vary substantially across ethnicities and geographic regions, necessitating population-specific thresholds [9]. This study was therefore designed to estimate the prevalence of MetS in an urban community of Jaipur using National Heart, Lung, and Blood Institute (NHLBI) criteria; describe sociodemographic correlates of MetS; quantify the association and correlation of NC with MetS and conventional anthropometric indices; and determine sex-specific optimal NC cut-off values for MetS prediction through receiver operating characteristic (ROC) analysis. The findings are intended to inform affordable, community-level screening strategies for MetS in urban India.

## Materials and methods

Study design and setting

This was a community-based, cross-sectional, observational study conducted in the field practice area of the Urban Health Training Centre (UHTC) attached to Sawai Man Singh (SMS) Medical College and Hospital, Jaipur, Rajasthan, India, the apex tertiary-care institution of Rajasthan. Data collection was conducted from April 9 to July 20, 2023.

Ethical approval

The study protocol was approved by the Institutional Research Review Board and the Ethics Committee of SMS Medical College, Jaipur (Rajasthan), India (IEC approval 116/MC/EC/2023, valid for the period 2021-2024). The actual data collection for this study was conducted from April 9 to July 20, 2023, which falls within the approved IEC period. Written informed consent was obtained from each participant prior to enrollment. Participant identities were anonymized throughout the study.

Sample size and sampling technique

The sample size was calculated using the formula

$$n = \frac{Z^2 PQ}{E^2}$$

assuming a MetS prevalence of 46.7% [10] at 95% confidence (Z = 1.96) and an acceptable error of 5%. This yielded a minimum sample of 382; incorporating a 5% attrition buffer, 400 participants were enrolled.

Using a systematic random sampling technique, one residential colony was randomly selected from the UHTC field area. Every alternate household was approached beginning with the first household selected by a simple random method, until 400 eligible participants were enrolled. If a household was closed or had no eligible resident, the next household was approached.

Inclusion and exclusion criteria

Adults aged 18-60 years who had resided in the study area for ≥6 months and provided written informed consent were included in the study. Pregnant women; individuals with a history of neck or abdominal surgery, malignancy, or thyroid disease; those on weight-reduction medications; amputees; bedridden individuals; and uncooperative subjects were excluded.

Data collection instruments

A pre-designed, pre-tested, semi-structured interview schedule was collected: sociodemographic data (age, sex, religion, caste, family type, education, occupation, socioeconomic status was derived as per the year 2023 modified Kuppuswami scale [11], marital status, dietary habits, personal habits); anthropometric measurements; and clinical and biochemical parameters for MetS.

Anthropometric measurements

Body weight was measured using a calibrated TANITA Ironman BC-554® scale (Tanita Corporation, Tokyo, Japan) (precision 100 g) with light clothing and no footwear. Height was measured using a standard stadiometer (SECA 213, SECA GmbH, Hamburg, Germany) with participants standing erect against a flat wall. Body mass index (BMI) was classified per WHO criteria [12] and was computed as 

$$\mathrm{BMI} = \frac{\text{weight (kg)}}{\mathrm{height}^2 \text{ (m}^2\mathrm{)}}$$

WC was measured midway between the lowest rib margin and iliac crest using an inelastic tape with the subject standing relaxed at end-normal expiration. WC was considered abnormal at ≥90 cm in males and ≥80 cm in females.

NC was measured in triplicate by a single examiner at the level of the thyroid cartilage (below the laryngeal prominence in males) with an inelastic tape, while subjects stood upright facing the horizon with shoulders relaxed. The modal value of the three measurements was recorded. NC ≥37 cm in males and ≥34 cm in females was considered abnormal per Bochaliya et al. [10].

Clinical and biochemical assessments

Blood pressure was measured in duplicate using an Omron HEM-7156 INT IntelliSense® (Omron Healthcare Co., Ltd., Kyoto, Japan) automatic sphygmomanometer after at least five minutes of seated rest; the mean of two readings was used. Hypertension was defined as systolic BP (SBP) ≥130 mmHg and/or diastolic BP (DBP) ≥85 mmHg, or current antihypertensive therapy.

Venous blood samples were collected after a minimum 12-hour overnight fast (home visits scheduled between 08:00 and 10:00 h). Plasma glucose was assayed by the glucose oxidase method [13]. A fasting plasma glucose (FPG) ≥100 mg/dL or current anti-diabetic therapy was considered abnormal. Lipid profile (total cholesterol, HDL-C, low-density lipoprotein cholesterol (LDL-C), triglycerides (TG)) was measured from the same fasting sample using standard enzymatic methods [14].

Diagnosis of metabolic syndrome

MetS was diagnosed per the 2022 NHLBI harmonized criteria [1], requiring the presence of three or more of the following five components: abdominal obesity (WC ≥90 cm in males, ≥80 cm in females); elevated TG (≥150 mg/dL or lipid-lowering therapy); reduced HDL-C (<40 mg/dL in males, <50 mg/dL in females, or specific drug treatment); elevated BP (SBP ≥130 mmHg and/or DBP ≥85 mmHg or antihypertensive therapy); and elevated fasting glucose (≥100 mg/dL or anti-diabetic therapy).

Statistical analysis

Data were entered into Microsoft Excel (Microsoft Corporation, Redmond, Washington, United States) and analyzed using IBM SPSS Statistics for Windows, Version 25 (Released 2017; IBM Corp., Armonk, New York, United States) (licensed). Categorical variables were expressed as frequencies and proportions; continuous variables as mean ± standard deviation (SD). The chi-square test was used to assess associations between categorical variables. Pearson's correlation coefficient was used to evaluate linear relationships between NC, WC, and BMI. Binary logistic regression was performed to identify independent predictors of MetS.

ROC curve analysis was employed to determine optimal NC cut-off values for MetS detection, with sensitivity, specificity, and diagnostic accuracy computed at each threshold. Diagnostic accuracy was calculated as

$$\mathrm{DA} = \frac{(TP + TN)}{N} \times 100$$

where TP = true positives, TN = true negatives, and N = total participants in the sex-specific subgroup. The optimal NC cut-off for each sex was identified by the Youden Index

$$J = \mathrm{Sensitivity} + \mathrm{Specificity} - 1$$

range 0-1; higher values indicate better overall diagnostic discrimination. A p-value of <0.05 was considered statistically significant.

## Results

Sociodemographic profile

As per Table 1, a total of 400 adults (207 females (51.8%) and 193 males (48.3%)) were enrolled. The mean age was 39.11 ± 12.7 years (range: 18-60 years) with a male-to-female ratio of 0.93. The age distribution was relatively even across groups: 18-30 years (n = 132, 33%), 31-40 years (n = 94, 23.5%), 41-50 years (n = 78, 19.5%), and 51-60 years (n = 96, 24%).

**Table 1:** Age-wise distribution of study participants (N = 400)

Age Groups	Females		Males		Total	
	Number	%	Number	%	Number	%
18-30 Years	70	53.0	62	47.0	132	33
31-40 Years	51	54.3	43	45.7	94	23.5
41-50 Years	38	48.7	40	51.3	78	19.5
51-60 Years	48	50.0	48	50.0	96	24
Grand Total	207	51.8	193	48.3	400	100

As per Table 2, Hindus comprised 72.7% (n = 291) of participants and Muslims 27.3% (n = 109). The majority (n = 234, 58.5%) lived in joint families. Education ranged from illiteracy (n = 38, 9.5%) to post-graduate level (n = 7, 1.7%); graduates constituted the largest group (n = 111, 27.8%). Nearly half (n = 195, 48.8%) were unemployed. According to the modified Kuppuswami socioeconomic scale, 98.5% (n = 394) belonged to the middle and upper-lower classes (Classes II-IV). Most participants (n = 323, 80.8%) were married, and 66% (n = 264) were vegetarians.

**Table 2:** Sociodemographic profile of study participants (N = 400) *Modified Kuppuswami socioeconomic scale (SES) Classification Year 2023 [11] OBC: other backward classes; SC: scheduled caste; ST: scheduled tribe

Religion	Number	Percentage (%)
Hindu	291	72.7
Muslims	109	27.3
Caste	Number	Percentage (%)
General	86	21.5
OBC	187	46.7
SC	63	15.8
ST	64	16
Family Type	Number	Percentage (%)
Nuclear	163	40.8
Joint	234	58.5
Extended	3	0.7
Educational Status	Number	Percentage (%)
Illiteracy	38	9.5
Primary	35	8.8
Middle	58	14.5
High School	81	20.2
Intermediate/Diploma	70	17.5
Graduate	111	27.8
Post-graduate/Professional	7	1.7
Occupational Status	Number	Percentage (%)
Unemployed	195	48.8
Elementary Occupation	46	11.5
Plant & Machinery related	19	4.7
Craft and Trade-related	65	16.2
Skilled Agriculture & Fishery	14	3.5
Shopkeepers	16	4
Clerk/Technicians/Associates	21	5.2
Post-graduate/Professional	22	5.5
Legislators/CEO/ Managerial	2	0.5
Socio-Economic Status*	Number	Percentage (%)
Class I	4	1
Class II	102	25.5
Class III	151	37.8
Class IV	141	35.2
Class V	2	0.5

Prevalence of metabolic diseases and risk factors

Table 3 summarizes the prevalence of metabolic diseases and key anthropometric parameters. An altered lipid profile (at least one abnormal lipid parameter) was the most prevalent metabolic disorder (n = 366, 91.5%), followed by obesity (BMI > 25 kg/m²) (n = 143, 35.8%), hypertension (n = 97, 24.3%), and diabetes (n = 69, 17.3%). Among the seven MetS-related investigational parameters, hypertriglyceridemia was the most common abnormality (n = 239, 59.8%), followed by elevated SBP (n = 97, 24.3%), low HDL-C (n = 91, 22.8%), elevated FPG (n = 69, 17.3%), elevated DBP (n = 67, 16.8%), elevated LDL-C (n = 40, 10%), and hypercholesterolemia (n = 23, 5.8%).

**Table 3:** Metabolic risk factor profile of study participants (N = 400) M: males; F: females; SBP: systolic blood pressure; DBP: diastolic blood pressure; HDL-C: high-density lipoprotein cholesterol; LDL-C: low-density lipoprotein cholesterol; BMI: body mass index

Parameter	Mean ± SD	Abnormal n (%)	Threshold (M)	Threshold (F)
BMI (kg/m²)	24.4 ± 3.1	143 (35.8%)	>25	>25
Waist Circumference (cm)	86.1 ± 7.4	153 (38.4%)	≥90	≥80
Neck Circumference (cm)	35.0 ± 3.1	134 (33.5%)	≥37	≥34
SBP (mmHg)	125.1 ± 16.5	97 (24.3%)	≥130	≥130
DBP (mmHg)	79.0 ± 9.8	67 (16.8%)	≥85	≥85
Fasting Blood Sugar (mg/dL)	95.2 ± 28.8	69 (17.3%)	≥100	≥100
Total Cholesterol (mg/dL)	157.6 ± 24.4	23 (5.8%)	N/A	N/A
HDL-C (mg/dL)	51.8 ± 5.6	91 (22.8%)	<40	<50
LDL-C (mg/dL)	86.7 ± 15.0	40 (10.0%)	N/A	N/A
Triglycerides (mg/dL)	165.9 ± 53.7	239 (59.8%)	≥150	≥150

Prevalence of metabolic syndrome

MetS was detected in 174 (43.5%) of the 400 participants using NHLBI criteria. Only six participants (1.5%) had none of the studied MetS risk factors. Two or more risk factors were present in 70.2% (n = 281) of participants; more than four were present in 22% (n = 88).

Sociodemographic correlates of metabolic syndrome

Table 4 summarizes the associations between sociodemographic variables and MetS. MetS prevalence increased progressively with age: 17.4% (18-30 years) (n = 23), 45.7% (31-40 years) (n = 43), 48.7% (41-50 years) (n = 38), and 72.9% (51-60 years) (n = 70) (p < 0.001). Females had a significantly higher prevalence than males (105/207 (50.7%) vs. 69/193 (35.7%), p = 0.004). MetS was significantly more common in married participants than unmarried ones (159/323 (49.2%) vs. 15/77 (19.5%), p < 0.001). Education, occupation, and socioeconomic status were also significantly associated (p < 0.05 each). Notably, MetS was more frequent at educational extremes (illiteracy (n = 31. 81.6%); postgraduate level (n = 6, 85.7%)) and among those in managerial roles (n = 2, 100%) and shopkeepers (n = 10, 62.5%) (Table 2). Alcohol consumption was associated with a higher MetS prevalence than tobacco smoking. Religion, caste, family type, and dietary habits were not significantly associated with MetS (all p > 0.05).

**Table 4:** Association of sociodemographic variables with metabolic syndrome (N = 400) *Statistically significant (p < 0.05). SES: socioeconomic status; NS: not significant; MetS: metabolic syndrome

Variable	MetS Absent n (%)	MetS Present n (%)	P-value
Age: 18–30 years	109 (82.6)	23 (17.4)	< 0.001*
Age: 31–40 years	51 (54.3)	43 (45.7)	
Age: 41–50 years	40 (51.3)	38 (48.7)	
Age: 51–60 years	26 (27.1)	70 (72.9)	
Sex: Female	102 (49.3)	105 (50.7)	0.004*
Sex: Male	124 (64.3)	69 (35.7)	
Religion: Hindu	160 (55.0)	131 (45.0)	0.375
Religion: Muslim	66 (60.6)	43 (39.4)	
Education (overall)	—	—	< 0.001*
Illiteracy	7 (18.4)	31 (81.6)	
Graduate	83 (74.8)	28 (25.2)	
Post-graduate	1 (14.3)	6 (85.7)	
Occupation (overall)	—	—	0.017*
SES (overall)	—	—	0.016*
Marital Status: Married	164 (50.8)	159 (49.2)	< 0.001*
Marital Status: Unmarried	62 (80.5)	15 (19.5)	
Personal Habits: Alcohol	17 (37.7)	28 (62.3)	0.027*
Type of Diet	—	—	0.618 (NS)

Association of anthropometric indices with metabolic syndrome

Both elevated BMI and abnormal WC were significantly associated with MetS (both p < 0.001). All 19 obese participants (BMI > 30 kg/m²) had MetS (100%), and 86.9% (n = 133) of those with abnormal WC had MetS, compared with only 16.6% (n = 41) with normal WC (chi-square = 187.28, p < 0.001).

Among the 134 participants with abnormal NC, 128 (95.5%) had MetS; among the 266 with normal NC, only 46 (17.3%) had MetS (chi-square = 218.71, p < 0.001) (Table 5). Binary logistic regression with NC as the sole independent variable confirmed NC as a statistically significant predictor of MetS on binary logistic regression in the overall study population (OR = 1.91 per 1 cm increase in NC; 95% CI 1.68-2.18; p < 0.001; McFadden Pseudo-R² = 0.36). Sex-stratified analysis revealed a stronger association in males (OR = 6.92; 95% CI 3.55-13.48; p < 0.001) compared to females (OR = 2.27; 95% CI 1.80-2.86; p < 0.001).

**Table 5:** Association of neck circumference with metabolic syndrome and binary logistic regression analysis of NC as a significant predictor of MetS (n = 400) *Statistically significant (p < 0.05). Normal NC: <37 cm (males), <34 cm (females). Abnormal NC: ≥37 cm (males), ≥34 cm (females). Chi-square (Part A) = 218.712, df = 1, p < 0.001. OR: odds ratio per 1 cm increment in NC (continuous variable); CI: confidence interval; NC: neck circumference; MetS: metabolic syndrome

Part A: Association of NC Category With Metabolic Syndrome				
NC Category	MetS Absent n (%)	MetS Present n (%)	Total	P-value
Normal NC (N = 266)	220 (82.7)	46 (17.3)	266	< 0.001*
Abnormal NC (N = 134)	6 (4.5)	128 (95.5)	134	
Total	226 (56.5)	174 (43.5)	400	
Part B: Binary Logistic Regression — NC as Significant Predictor of MetS				
Predictor (NC)	N	Odds Ratio	95% Confidence Interval	p-Value
NC (Overall)	400	1.913	1.679 - 2.181	< 0.001*
NC (Males)	193	6.919	3.553 - 13.476	< 0.001*
NC (Females)	207	2.271	1.803 - 2.860	< 0.001*

Correlation of neck circumference with other anthropometric indices

NC showed a strong positive correlation with WC (r = 0.705, p < 0.001; R² = 0.497), with the regression equation:

$$\mathrm{WC} = 27.901 + 1.663 \times \mathrm{NC}$$

 NC also demonstrated a significant positive correlation with BMI (r = 0.581, p < 0.001; R² = 0.337), with the equation:

$$\mathrm{BMI} = 4.568 + 0.565 \times \mathrm{NC}$$

 For each 1 cm increase in NC, WC increased by approximately 1.663 cm and BMI by 0.565 kg/m².

Optimal NC cut-off values for metabolic syndrome

ROC curve analysis was performed separately for males and females. Table 6, 7 presents diagnostic performance metrics at key NC thresholds.

**Table 6:** ROC analysis-NC cut-off values for MetS in males (N = 193; MetS present = 69, MetS absent = 124) *Optimal cut-off: NC >=37 cm (Youden Index J = 0.830 = maximum). Sensitivity 87.0%, specificity 96.0%, diagnostic accuracy 92.7%. AUC = 0.946. NC: neck circumference; MetS: metabolic syndrome; ROC: receiver operating characteristic Youden Index$J = \mathrm{Sensitivity} + \mathrm{Specificity} - 1$ (range 0-1; maximum identifies optimal threshold)$\mathrm{DA} = \frac{(TP + TN)}{N} \times 100$. $\mathrm{FPR} = 1 - \mathrm{Specificity}$. $\text{Avg Sn+Sp} = \frac{(\mathrm{Sensitivity} + \mathrm{Specificity})}{2}$

NC (cm)	Sn (%)	Sp (%)	FPR (1-Sp)	Avg Sn+Sp	DA (%)	Youden Index (J)	AUC
34	100.0	20.2	0.798	60.1	48.7	0.202	
35	100.0	45.2	0.548	72.6	64.8	0.452	
36	94.2	71.0	0.290	82.6	79.3	0.652	
>=37 (optimal)*	87.0	96.0	0.040	91.5	92.7	0.830	0.946
38	75.4	98.4	0.016	86.9	90.2	0.738	
40	44.9	99.2	0.008	72.0	79.8	0.441	

**Table 7:** ROC analysis-NC cut-off values for MetS in females (N = 207; MetS present = 105, MetS absent = 102) *Optimal cut-off: NC >=34 cm (Youden Index J = 0.636 = maximum). Sensitivity 71.4%, specificity 92.2%, diagnostic accuracy 81.6%. AUC = 0.872. NC: neck circumference; MetS: metabolic syndrome; ROC: receiver operating characteristic Note on identical FPR at NC = 35 and NC = 36 (0.010): This is mathematically valid; it indicates no MetS-negative female in the dataset has NC in the 35.0-35.9 cm range, so the false positive count does not change when the threshold rises from 35 to 36 cm. Youden Index $J = \mathrm{Sensitivity} + \mathrm{Specificity} - 1$. $\mathrm{DA} = \frac{(TP + TN)}{N} \times 100$. $\mathrm{FPR} = 1 - \mathrm{Specificity}$

NC (cm)	Sn (%)	Sp (%)	FPR (1-Sp)	Avg Sn+Sp	DA (%)	Youden Index (J)	AUC
32	97.1	35.3	0.647	66.2	66.7	0.324	
33	88.6	60.8	0.392	74.7	74.9	0.494	
>=34 (optimal)*	71.4	92.2	0.078	81.8	81.6	0.636	0.872
35	58.1	99.0	0.010	78.6	78.3	0.571	
36	42.9	99.0	0.010	71.0	70.5	0.419	
37	29.5	100.0	0.000	64.8	64.3	0.295	

The ROC analysis confirmed NC ≥37 cm as the optimal cut-off for males and NC ≥34 cm for females, representing the best balance of sensitivity and specificity for MetS prediction. The optimal thresholds align precisely with the Bochaliya et al. [10] reference cut-offs, substantially strengthening their external validity in the North Indian urban context.

## Discussion

This community-based study from urban Jaipur documents a high prevalence of MetS (43.5%) in adults aged 18-60 years, with NC emerging as a robust, low-cost, easily replicable screening tool strongly associated with MetS and correlated with established anthropometric indices.

Prevalence of metabolic syndrome

The MetS prevalence of 43.5% in our study is consistent with published figures from urban North Indian cohorts. Bochaliya et al. [10] reported 46.7% in Western India; Pavithra, H. et al. [15] reported a prevalence of MS of 33.9%, and the majority were females (71.8%). Age-adjusted prevalence of metabolic syndrome was found to be overall approximately 25% by Bhalwar R et al. [16], and Khan et al. [17] observed 41% in Uttar Pradesh. Our figure is higher than reported from a rural Rajasthan-based study (16-29%) [18], reinforcing the established urban-rural disparity in MetS burden, possibly attributable to differences in physical activity, dietary patterns, and stress levels.

Dyslipidemia (91.5%) was the predominant metabolic abnormality, consistent with data from a study [19], who found 79% of their urban Indian sample had at least one lipid abnormality. Hypertriglyceridemia (59.8%) was the single most frequent component in our study, mirroring findings from Indian Council of Medical Research-INdia DIABetes (ICMR-INDIAB) data [20] and aligning with the increasingly recognized role of postprandial lipemia in Indian populations consuming high-carbohydrate diets.

Sociodemographic correlates of metabolic syndrome

The significant age gradient in MetS prevalence (17.4% in the youngest to 72.9% in the oldest age group, p < 0.001) is consistent with accumulating evidence that metabolic deterioration is an aging-related phenomenon influenced by declining insulin sensitivity, physical activity, and gonadal hormone levels [21]. The higher MetS prevalence in females (50.7% vs. 35.7%, p = 0.004) has been documented across Indian studies and is likely attributable to the postmenopausal hormonal milieu, greater central adiposity accrual in Indian women, and higher rates of physical inactivity among urban women [16].

The finding that MetS was more common at educational extremes, highest among the illiterate (81.6%) and postgraduates (85.7%), is intriguing and likely reflects divergent socioeconomic and occupational pathways rather than a single mechanistic explanation. Among individuals with lower educational attainment, reduced access to preventive healthcare, and limited health literacy may contribute to delayed recognition and management of metabolic risk factors. Among those with higher educational attainment, sedentary professional occupations and psychosocial stress may be the dominant drivers. Importantly, dietary quality and physical activity patterns cannot be attributed uniformly to any single educational stratum, as both behaviors are shaped by a complex interplay of cultural norms, occupational demands, economic access, and individual choice across all literacy levels. Alcohol consumption was associated with higher MetS prevalence than tobacco use, consistent with the established lipogenic and blood-pressure-elevating effects of alcohol [22].

NC as an indicator of metabolic syndrome

The highly significant association between abnormal NC and MetS (chi-square = 218.71, p < 0.001) is the cardinal finding of this study. The observation that 95.5% of those with abnormal NC had MetS, against only 17.3% of those with normal NC, underscores the clinical utility of NC as a high-specificity flag for MetS risk. This strong association has been corroborated by several investigators: Bochaliya et al. [10] found a significant association (p < 0.001) in a Western Indian hospital cohort; Kumar NV et al. [23] noted 77.7% of MetS subjects had elevated NC; and Issarayus et al. [24] reported significantly higher mean NC in MetS versus non-MetS individuals across a Southeast Asian population.

The correlations of NC with WC (r = 0.705) and BMI (r = 0.581), both highly significant, highlight that NC captures adiposity-related variance in WC and BMI but is neither identical nor redundant with these measures. Several studies support this complementarity: Raimi et al. [25] found NC correlated with WC, BMI, and SBP; Joshipura et al. [26] demonstrated NC was superior or equivalent to WC for predicting multiple cardiometabolic risk factors, and Luo et al. [27] showed NC was non-inferior to WC in detecting MetS using imaging-validated body composition. The practical advantage of NC over WC is particularly relevant in field settings: NC measurement avoids issues of undressing and is less influenced by bloating or fecal loading, making it consistently reproducible across varying field conditions.

NC cut-off values

The ROC-derived optimal NC thresholds of 37 cm in males (sensitivity 87.0%, specificity 96.0%, Avg Sn+Sp 91.5%, diagnostic accuracy 92.7%) and 34 cm in females (sensitivity 71.4%, specificity 92.2%, Avg Sn+Sp 81.8%, diagnostic accuracy 81.6%) are consistent with Indian data and lower than those reported from Western, African, and Middle Eastern populations, reflecting the lower absolute anthropometric thresholds characterizing the lean-obese metabolic phenotype prevalent in South Asian individuals. Notably, the data-derived optimal thresholds independently corroborate the Bochaliya et al. [10] reference cut-offs (37 cm males, 34 cm females), substantially strengthening the external validity of both thresholds. Within India, Kumar et al. [28] found 38 cm and 34.7 cm in a central Indian cohort; Mondal et al. [29] proposed 36.25 cm and 32.5 cm in Kolkata.

Both the male and female optimal cut-offs demonstrate high specificity. The male cut-off (NC ≥ 37 cm) delivers sensitivity 87.0%, specificity 96.0%, and diagnostic accuracy 92.7% performance metrics that exceed the conventional 80% threshold across all three parameters, positioning NC as an excellent, well-rounded screening tool in males. The female cut-off (NC ≥ 34 cm) demonstrates a specificity-dominant profile (specificity 92.2%, sensitivity 71.4%, diagnostic accuracy 81.6%), conferring strong screen-positive predictive value: when the test is positive, it is unlikely to be a false alarm, which is operationally useful in community settings where unnecessary confirmatory biochemical workup imposes logistical and financial burden. 

A comparison of NC cut-off values from the current study with published data from similar populations is provided in Table 8.

**Table 8:** Comparison of NC cut-off values for MetS across studies NHLBI: National Heart, Lung, and Blood Institute; NCEP-ATP III: National Cholesterol Education Program Adult Treatment Panel III; IDF: International Diabetes Federation; NC: neck circumference; MetS: metabolic syndrome

Study (Year)	Setting/Country	N	Male (cm)	Female (cm)	Criteria
Present Study (2023)	Jaipur, India	400	37	34	NHLBI
Bochaliya RK et al. (2019) [10]	West India	405	37	34	NHLBI
Murthy SSN et al. (2019) [30]	South India	512	36	29	Community
Kumar S et al. (2012) [28]	Wardha, India	203	38	34.7	Community
Mondal SA et al. (2018) [29]	Kolkata, India	188	36.25	32.5	Community
Issarayus L et al. (2020) [24]	Bangkok, Thailand	390	38	33	NCEP-ATP III
Ebrahimi H et al. (2016) [9]	Iran	1619	42	36	IDF
Raimi TH et al. (2021) [25]	Nigeria	557	40	33	IDF

Clinical and public health implications

These findings have direct relevance for primary and community healthcare. NC measurement requires only an inexpensive, widely available inelastic tape, takes less than 30 seconds, requires no privacy, and involves no discomfort. Incorporating NC screening into routine health-worker assessments at urban primary health centers, anganwadis, and UHTC outreach camps could enable rapid, cost-effective flagging of high-risk adults for further MetS evaluation. Given the high MetS prevalence (43.5%) in this community, there is an urgent need to scale up preventive interventions targeting dietary modification, physical activity promotion, and alcohol reduction in urban Rajasthan.

Limitations

This study has limitations. First, as a cross-sectional design, causality cannot be inferred. Second, the study was confined to a single urban UHTC area in Jaipur, limiting generalizability to rural populations. Third, the relatively small sample size for subgroup analyses may limit power for detecting modest associations. Fourth, physical activity and dietary intake data were collected by self-report, subject to recall bias.

## Conclusions

This community-based study demonstrates that MetS is highly prevalent in urban Jaipur, underscoring the urgent need for scalable, affordable screening strategies in resource-constrained Indian healthcare settings.

NC emerges as a powerful, statistically significant, and easily applicable anthropometric tool that is strongly correlated with established obesity indicators, making it well-suited for rapid MetS risk flagging in primary care and community health worker programs. Sex-specific optimal NC thresholds were identified for this North Indian urban population, offering a clinically useful balance between sensitivity and specificity.

The key message of this study is that a simple tape-measure assessment of the neck requiring no laboratory infrastructure, no undressing, and less than one minute can substantially improve early MetS detection at the population level. Integrating NC screening into routine health assessments at urban primary health centers, anganwadis, and outreach camps could support timely preventive interventions targeting dietary modification, physical activity promotion, and cardiovascular risk reduction in urban India. Replication in larger, longitudinal, multi-site studies using South Asian-specific consensus criteria is recommended.
